# Antecedent glucagon-like peptide-1 receptor agonist use and risk of sepsis-induced cardiomyopathy in type 2 diabetes: a United States real-world active-comparator cohort study

**DOI:** 10.3389/fphar.2026.1877392

**Published:** 2026-07-31

**Authors:** Jheng-Yan Wu, Keng-Wei Lee, Sheng-Chi Huang, Hsuan-Yuan Chang, Yu-Min Lin

**Affiliations:** 1 Department of Nutrition, Chi Mei Medical Center, Tainan, Taiwan; 2 Department of Public Health, College of Medicine, National Cheng Kung University, Tainan, Taiwan; 3 Division of Cardiology, Department of Internal Medicine, Chi Mei Hospital, Chiali, Tainan, Taiwan; 4 Department of Medical Education, Chi Mei Medical Center, Tainan, Taiwan; 5 Division of Hepatogastroenterology, Department of Internal Medicine, Chi Mei Medical Centre, Tainan, Taiwan; 6 Division of Cardiology, Department of Internal Medicine, Chi Mei Medical Center, Tainan, Taiwan

**Keywords:** dipeptidyl peptidase-4 inhibitor, glucagon-like peptide-1 receptor agonist, real-world evidence, sepsis-induced cardiomyopathy, type 2 diabetes mellitus

## Abstract

**Background:**

Sepsis-induced cardiomyopathy (SICM) is an important cardiovascular complication of infection and sepsis, particularly in patients with type 2 diabetes mellitus (T2DM). Whether antecedent glucagon-like peptide-1 receptor agonist (GLP-1 RA) use is associated with lower SICM risk remains unclear.

**Methods:**

We conducted a retrospective active-comparator cohort study using the TriNetX United States federated electronic health record network from 1 January 2010, to 30 November 2025. Adults with T2DM and documented infection were classified according to antecedent GLP-1 RA or dipeptidyl peptidase-4 inhibitor (DPP-4i) exposure before the index infection date and matched 1:1 by propensity score. The primary outcome was 1-year EHR-ascertained SICM or SICM-related cardiac dysfunction, defined using diagnostic codes for acute pulmonary edema, heart failure, cardiogenic shock, or cardiomyopathy, or objective cardiac dysfunction.

**Results:**

Among 189,156 eligible patients, propensity-score matching yielded 62,267 patients in each group. Over 1 year, EHR-ascertained SICM or SICM-related cardiac dysfunction occurred in 2,503 patients (4.0%) in the GLP-1 RA group and 3,059 patients (4.9%) in the DPP-4i group (HR 0.82, 95% CI 0.78–0.87; P < 0.001; E-value 1.7). GLP-1 RA use was also associated with lower risks of surrogate cardiac dysfunction (HR 0.70, 95% CI 0.64–0.76), systolic SICM (HR 0.85, 95% CI 0.77–0.93), diastolic SICM (HR 0.88, 95% CI 0.81–0.95), hyperdynamic SICM (HR 0.82, 95% CI 0.71–0.95), new-onset heart failure (HR 0.85, 95% CI 0.80–0.89), and all-cause mortality (HR 0.64, 95% CI 0.60–0.68), but not right ventricular dysfunction SICM. Negative control outcomes showed null associations, and landmark analyses were consistent.

**Conclusion:**

In this United States real-world active-comparator cohort study, antecedent GLP-1 RA exposure was associated with a lower 1-year risk of EHR-ascertained SICM or SICM-related cardiac dysfunction compared with antecedent DPP-4i exposure. These findings were consistent across sensitivity analyses and support further prospective investigation into the relationship between antecedent GLP-1 RA exposure and cardiovascular vulnerability after infection.

## Introduction

Infections remain one of the leading causes of hospitalization worldwide and impose a substantial burden on healthcare systems, particularly among individuals with chronic cardiometabolic conditions (Global and regional, 2025). In a substantial proportion of patients, infections progress to sepsis, a life-threatening syndrome characterized by dysregulated host responses and multi-organ dysfunction, with persistently high mortality (Meyer and Prescott, 2024). Beyond acute morbidity and mortality, sepsis is frequently complicated by cardiovascular dysfunction, including sepsis-induced cardiomyopathy (SICM), has been consistently associated with adverse short- and long-term prognosis (Lai et al., 2025; Hasegawa et al., 2023; Hanumanthu et al., 2021; Lin et al., 2022).

Patients with type 2 diabetes mellitus (T2DM) are disproportionately affected by sepsis and experience worse clinical outcomes compared with those without diabetes, a disparity that may be partly attributable to a heightened vulnerability to cardiovascular injury during acute infection arising from underlying metabolic and vascular derangements (Holt et al., 2024; Aissaoui et al., 2025). Chronic hyperglycemia and insulin resistance are associated with immune dysregulation, systemic inflammation, and endothelial dysfunction, which may exacerbate hemodynamic and inflammatory stress during infection or sepsis (Jiang and Cheng, 2022; Costantini et al., 2021). Moreover, individuals with T2DM frequently have subclinical myocardial dysfunction and a high burden of cardiometabolic comorbidities, rendering the myocardium less resilient to acute inflammatory and circulatory insults (Nghiem et al., 2025; Wong et al., 2023). Collectively, these features provide a biologically plausible substrate through which infection-related systemic inflammation may translate into myocardial injury and transient cardiac dysfunction.

Glucagon-like peptide-1 receptor agonists (GLP-1 RAs) are widely prescribed glucose-lowering therapies for patients with T2DM and are frequently used in populations at high risk for infection and sepsis (Razavi et al., 2022). Beyond glycemic control, accumulating evidence suggests that GLP-1 RAs exert pleiotropic effects, including anti-inflammatory properties, modulation of innate immune responses, and improvements in endothelial function (C et al., 2022; Zheng et al., 2024). Recent observational and experimental studies have further suggested that GLP-1 RA use may be associated with a lower risk of certain infections or infection-related complications (Han et al., 2025; Wang et al., 2025). Against this background, it remains unclear whether pre-infection use of GLP-1 RAs is associated with a differential risk of SICM in T2DM.

Accordingly, we conducted a United States real-world cohort study using the TriNetX federated electronic health record network to compare patients with T2DM receiving GLP-1 RAs versus dipeptidyl peptidase-4 inhibitors (DPP-4is) prior to an index infection. The primary outcome was the 1-year risk of SICM, with secondary outcomes including new-onset heart failure and all-cause mortality. We further characterized distinct phenotypes of SICM, including left ventricular systolic dysfunction (LVSD), left ventricular diastolic dysfunction (LVDD), right ventricular injury (RVI), and left ventricular hyperdynamic function (LVHF).

## Methods

### Data source

This retrospective cohort study was conducted using data from the TriNetX research network, a federated electronic health record platform encompassing approximately 113 million patients across 68 healthcare organizations within the United States network. The database provides access solely to aggregated and deidentified data, including information on clinical diagnoses, laboratory measurements, procedures, medication prescriptions, and genomic characteristics. Investigators are not granted access to protected health information and are unable to identify or directly contact individual patients. Because the dataset contains no direct personal identifiers and does not involve direct patient interaction, review and approval by the Western Institutional Review Board were not required. The study was designed, conducted, and reported in accordance with the Strengthening the Reporting of Observational Studies in Epidemiology guidelines (Cuschieri, 2019).

### Study design

Adults aged 18 years or older with a documented diagnosis of infection and T2DM between 1 January 2010, and 30 November 2025, were identified (Table 1). Infection and T2DM were defined using ICD-10-CM codes A00–B99 and E11, respectively. Eligible patients were categorized into two exposure groups. The index date was defined as the date of the first documented infection during the study period. Antecedent exposure was defined as at least one prescription record for a GLP-1 RA or DPP-4i during the 12-month period before or on the index infection date. To maintain mutual exclusivity between groups, individuals were additionally excluded if they had received the alternative study medication within 3 months before the index date. For example, patients in the GLP-1RA group were excluded if they had received a DPP-4i within 3 months before or after initiation of GLP-1RA therapy. An active-comparator exposure design was used to compare patients with antecedent GLP-1 RA exposure with those with antecedent DPP-4i exposure before the index infection date. This design was intended to reduce confounding by indication by selecting an alternative glucose-lowering medication class as the comparator and by aligning baseline covariate assessment before the index infection date. Patients exposed to both study drug classes during the baseline exposure window were excluded to maintain mutually exclusive comparison groups. Detailed definitions of variables and coding algorithms are provided in Supplementary Table S1.

**TABLE 1:** Baseline characteristics of GLP-1RA and DPP-4i groups before and after matching.

Variables	Before matching			After matching		
	GLP-1RA group (n = 111,147)	DPP-4i group (n = 78,009)	Standardized difference	GLP-1RA group (n = 62,267)	DPP-4i group (n = 62,267)	Standardized difference
Age, years						
Mean (SD)	57.9 (12.7)	64.7 (13)	0.534	61.9 (11.7)	62 (12.4)	0.009
Sex, n (%)						
Female	63,747 (57.4)	40,271 (51.6)	0.115	33,135 (53.2)	33,189 (53.3)	0.002
Male	47,353 (42.6)	37,700 (48.3)	0.115	29,100 (46.7)	29,046 (46.6)	0.002
Race, n (%)						
White	75,068 (67.5)	49,783 (63.8)	0.078	41,272 (66.3)	41,374 (66.4)	0.003
Black or African American	20,467 (18.4)	13,781 (17.7)	0.019	11,061 (17.8)	11,245 (18.1)	0.008
Asian	3,619 (3.3)	5,304 (6.8)	0.163	2,805 (4.5)	2,602 (4.2)	0.016
Comorbidities, n (%)						
Overweight and obesity	42,930 (38.6)	15,784 (20.2)	0.412	15,425 (24.8)	15,471 (24.8)	0.002
Nicotine dependence	8,025 (7.2)	5,540 (7.1)	0.005	4,376 (7)	4,592 (7.4)	0.013
Dyslipidemia	70,203 (63.2)	47,317 (60.7)	0.052	38,008 (61)	38,222 (61.4)	0.007
Chronic kidney disease	12,379 (11.1)	12,425 (15.9)	0.14	8,453 (13.6)	8,455 (13.6)	0
Atrial fibrillation and flutter	4,113 (3.7)	4,312 (5.5)	0.087	2,805 (4.5)	2,849 (4.6)	0.003
T2DM with kidney complications	13,994 (12.6)	11,036 (14.1)	0.046	8,160 (13.1)	8,211 (13.2)	0.002
T2DM with ophthalmic complications	7,103 (6.4)	4,446 (5.7)	0.029	3,642 (5.8)	3,720 (6)	0.005
T2DM with neurological complications	17,755 (16)	11,074 (14.2)	0.05	9,096 (14.6)	9,371 (15.1)	0.012
T2DM with circulatory complications	6,641 (6)	4,027 (5.2)	0.035	3,292 (5.3)	3,366 (5.4)	0.005
Medications, n (%)						
ACE inhibitors	32,861 (29.6)	25,998 (33.3)	0.081	20,089 (32.3)	19,938 (32)	0.005
Angiotensin II inhibitors	27,224 (24.5)	19,026 (24.4)	0.002	14,950 (24)	15,202 (24.4)	0.009
Beta blockers	30,255 (27.2)	26,252 (33.7)	0.14	18,967 (30.5)	19,099 (30.7)	0.005
Biguanides	59,094 (53.2)	44,197 (56.7)	0.07	35,054 (56.3)	34,776 (55.9)	0.009
SGLT2is	24,879 (22.4)	11,040 (14.2)	0.214	10,723 (17.2)	10,406 (16.7)	0.014
Thiazolidinediones	5,244 (4.7)	5,482 (7)	0.098	3,691 (5.9)	3,703 (5.9)	0.001
Insulins	43,365 (39)	27,547 (35.3)	0.077	22,554 (36.2)	22,655 (36.4)	0.003
eGFR, mL/min/1.73m2						
Mean (SD)	81.3 (28.4)	74 (30.5)	0.246	77.5 (27.6)	76.2 (30.8)	0.042
≥45, n (%)	79,018 (71.1)	52,554 (67.4)	0.081	42,094 (67.6)	42,449 (68.2)	0.012
Hemoglobin A1c, %	​	​	​	​	​	​
Mean (SD)	7.7 (1.9)	7.8 (1.8)	0.045	7.7 (1.8)	7.9 (1.9)	0.088
≥9, n (%)	24,490 (22)	14,740 (18.9)	0.078	12,697 (20.4)	12,587 (20.2)	0.004
Lactate, mmol/L						
Mean (SD)	1.6 (0.9)	1.6 (0.9)	0.011	1.6 (0.8)	1.5 (0.9)	0.108
≥2, n (%)	2,207 (2)	2,534 (3.2)	0.079	1,523 (2.4)	1,561 (2.5)	0.004
Cholesterol in LDL, mg/dL	​	​	​	​	​	​
Mean (SD)	87.2 (37.1)	85.8 (36.8)	0.038	84.6 (36.8)	87.2 (36.9)	0.069
≥160, n (%)	3,568 (3.2)	2,254 (2.9)	0.019	1,879 (3)	1,897 (3)	0.002
Body mass index, kg/m2						
Mean (SD)	35.6 (8.0)	31.7 (7.4)	0.503	33.7 (7.6)	32.8 (7.4)	0.118
≥30, n (%)	61,544 (55.4)	29,905 (38.3)	0.347	27,516 (44.2)	27,794 (44.6)	0.009

ACE, angiotensin-converting enzyme; DPP-4i, dipeptidyl peptidase 4 inhibitors; eGFR, estimated Glomerular filtration rate; GLP-1, RA, glucagon-like peptide-1, receptor agonist; LDL, low-density lipoprotein; SD, standard deviation; SGLT2i, sodium-glucose co-transporter 2 inhibitor; T2DM, type 2 diabetes mellitus.

### Covariates and propensity score matching

After cohort identification and specification of index dates, outcomes, and potential confounders, baseline covariates were ascertained from data recorded during the 12 months preceding the index date. Propensity scores were estimated using multivariable logistic regression to calculate each patient’s probability of receiving the study treatment conditional on baseline characteristics measured prior to treatment initiation. Patients were subsequently matched in a 1:1 ratio using a greedy nearest-neighbor algorithm without replacement. A caliper width equal to 0.1 of the pooled standard deviation of the logit of the propensity score was applied to minimize poor matches. Covariate balance between treatment groups was assessed using standardized mean differences, with values less than 0.1 considered indicative of adequate balance in accordance with established recommendations (Haukoos and Lewis, 2015).

Baseline covariates included in the propensity score matching (PSM) were prespecified based on clinical relevance and prior literature to account for demographic characteristics, comorbid conditions, medication use, and key laboratory and physiological parameters that may influence treatment allocation and outcomes (Wu et al., 2026; Wu et al., 2025a; Wu et al., 2025b; Li et al., 2025). Age was summarized as mean ± SD. Sex was reported as n (%), categorized as female and male. Race was classified as White, Black or African American, and Asian, and presented as n (%). Comorbidities were expressed as n (%) and included overweight and obesity, nicotine dependence, dyslipidemia, CKD, atrial fibrillation and flutter, T2DM with kidney complications, T2DM with ophthalmic complications, T2DM with neurological complications, and T2DM with circulatory complications. Medication use at baseline was incorporated into the matching procedure and summarized as n (%). These medications included angiotensin-converting enzyme inhibitors, angiotensin II receptor blockers, beta blockers, biguanides, sodium-glucose cotransporter 2 (SGLT2) inhibitors, thiazolidinediones, and insulins. Continuous laboratory and physiological variables were reported as mean ± SD, including estimated glomerular filtration rate (eGFR; mL/min/1.73 m^2^), hemoglobin A1c (HbA1c; %), lactate (mmol/L), low-density lipoprotein cholesterol (mg/dL), and body mass index (BMI; kg/m^2^). Clinically relevant thresholds were additionally evaluated as categorical variables, defined as eGFR ≥45 mL/min/1.73 m^2^, HbA1c ≥9%, lactate ≥2 mmol/L, LDL (low-density lipoprotein) cholesterol ≥160 mg/dL, and BMI ≥30 kg/m^2^. Detailed variable definitions and coding criteria are provided in Supplementary Table S2. Available markers related to infection severity, including lactate level, lactate ≥2 mmol/L, and infection subtype, were incorporated into the adjustment strategy when available. However, granular critical care severity measures such as SOFA score, vasopressor use, ICU admission, mechanical ventilation, and source-control timing were not consistently available in the TriNetX dataset.

### Outcomes and follow-up

The primary endpoint was EHR-ascertained SICM or SICM-related cardiac dysfunction after the index infection date. This composite outcome was defined as the first post-index occurrence of acute pulmonary edema (ICD-10-CM J81.0), heart failure (I50), cardiogenic shock (R57.0), cardiomyopathy (I42), or objective evidence of cardiac dysfunction. Objective cardiac dysfunction was defined as at least one of the following post-index findings: left ventricular ejection fraction (LVEF) ≤50%, N-terminal pro–B-type natriuretic peptide (NT-proBNP) ≥1800 pg/mL, or cardiac troponin I ≥ 0.50 ng/mL. To reduce misclassification of chronic or prevalent cardiac disease as incident SICM, patients with any record of heart failure, cardiomyopathy, cardiogenic shock, acute pulmonary edema, or objective cardiac dysfunction before the index infection date were excluded from the corresponding outcome ascertainment. Because no universally accepted EHR-based diagnostic algorithm for SICM exists, we used a prespecified operational definition combining post-index diagnostic codes for acute pulmonary edema, heart failure, cardiogenic shock, or cardiomyopathy with objective evidence of cardiac dysfunction. Echocardiographic and biomarker criteria were used to capture cardiac dysfunction or myocardial injury in the EHR and were not intended to represent independently validated SICM-specific diagnostic criteria.

Detailed diagnostic codes, laboratory thresholds, and echocardiographic criteria are provided in Supplementary Table S3 (Wu et al., 2025c). Secondary outcomes included surrogate outcome, systolic type SIC, diastolic type SIC, right ventricular dysfunction type SIC, hyperdynamic type SIC, new-onset heart failure, and all-cause mortality. The surrogate outcome was defined by the presence of objective cardiac dysfunction, including LVEF ≤50%, NT-proBNP ≥1800 pg/mL, or cardiac troponin I ≥ 0.50 ng/mL, in the absence of the full SIC diagnostic criteria. Follow-up for study outcomes began on the day after the index date and continued until the first occurrence of the outcome of interest, the last recorded clinical encounter, death, or 1 year after cohort entry, whichever occurred first. Detailed outcome definitions and corresponding coding algorithms are provided in Supplementary Table S3.

### Subgroup analysis

For the primary outcome, prespecified subgroup analyses were conducted in accordance with the analytical plan. Patients were stratified by age (18–64 years vs. ≥ 65 years), sex, CKD stage (stage 3, stage 4, and stage 5), obesity status (BMI <30 vs. ≥ 30 kg/m^2^), and presence or absence of coronary artery disease (CAD). In addition, analyses were performed according to infection subtype, including skin infection, urinary tract infection, pneumonia, bacteremia, and sepsis.

### Sensitivity analysis

To evaluate the robustness of the study findings, hearing loss and hernia were examined as negative control outcomes, as these conditions are unlikely to be causally related to the study exposure. In addition, to mitigate potential time-dependent confounding inherent to this observational design, a landmark analysis was performed using a prespecified fixed time point after the index date.

### Statistical analysis

Continuous variables were summarized as mean ± standard deviation, whereas categorical variables were presented as counts and percentages. PSM was applied to balance baseline characteristics between treatment groups prior to the primary, subgroup, and sensitivity analyses. Hazard ratios (HRs) with 95% confidence intervals (CIs) were estimated using Cox proportional hazards regression models. Time-to-event outcomes were evaluated using Kaplan-Meier survival analysis, and between-group differences were assessed with the log-rank test. To quantify the potential impact of unmeasured confounding, E-values were calculated (VanderWeele and Ding, 2017). All statistical analyses were performed using the TriNetX research platform.

## Results

### Study cohort

**FIGURE 1:**
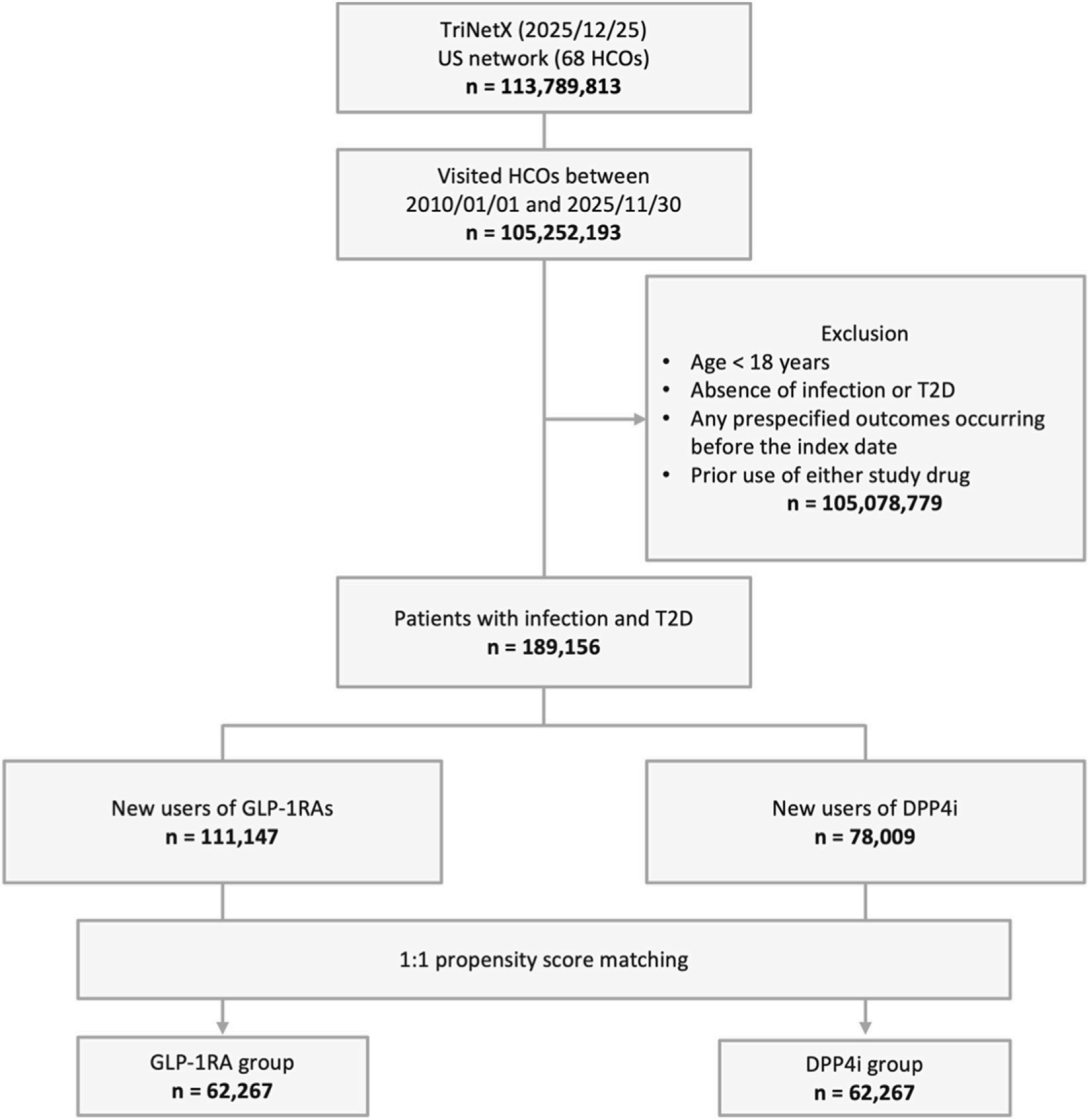
Study cohort process. DPP-4i, dipeptidyl peptidase-4 inhibitor; GLP-1 RA, glucagon-like peptide-1 receptor agonist; HCOs, healthcare organizations.

105,252,193 patients had at least one healthcare encounter between 1 January 2010, and 30 November 2025. After applying the exclusion criteria, 189,156 patients with both infection and T2DM were eligible for inclusion. Within this cohort, 111,147 patients were identified as having antecedent GLP-1 RA exposure and 78,009 patients as having antecedent DPP-4i exposure. After 1:1 PSM, two well-balanced cohorts were generated, each comprising 62,267 patients in the GLP-1RA and DPP-4i groups, respectively (Figure 1).

### Characteristics of study subjects

Before PSM, 111,147 patients in the GLP-1RA group and 78,009 patients in the DPP-4i group were identified. Several baseline characteristics differed substantially between groups. Patients receiving GLP-1RA were younger than those receiving DPP-4i (mean age 57.9 vs. 64.7 years; standardized difference 0.534). The GLP-1RA group also had a higher prevalence of overweight or obesity (38.6% vs. 20.2%; standardized difference 0.412) and greater use of SGLT2 inhibitors (22.4% vs. 14.2%; standardized difference 0.214). Differences were additionally observed in CKD, beta blocker use, and eGFR levels. After 1:1 matching, 62,267 patients remained in each group. Baseline characteristics were well balanced between groups, with all standardized differences below 0.1. The mean age was comparable between the GLP-1RA and DPP-4i groups (61.9 vs. 62.0 years; standardized difference 0.009). Sex distribution was similar, with 53.2% and 53.3% female patients, respectively. Comorbidities, including CKD, dyslipidemia, atrial fibrillation and flutter, and diabetes-related complications, were evenly distributed between groups. Baseline medication use, including ACE inhibitors, angiotensin II inhibitors, beta blockers, biguanides, SGLT2 inhibitors, thiazolidinediones, and insulin, was also well balanced. Laboratory and physiological parameters, including eGFR, HbA1c, lactate, LDL cholesterol, and BMI, demonstrated adequate balance after matching. The proportion of patients with eGFR ≥45 mL/min/1.73 m^2^, HbA1c ≥ 9%, lactate ≥2 mmol/L, LDL cholesterol ≥160 mg/dL, and BMI ≥30 kg/m^2^ was comparable between groups.

### Primary and secondary outcomes

**FIGURE 2:**
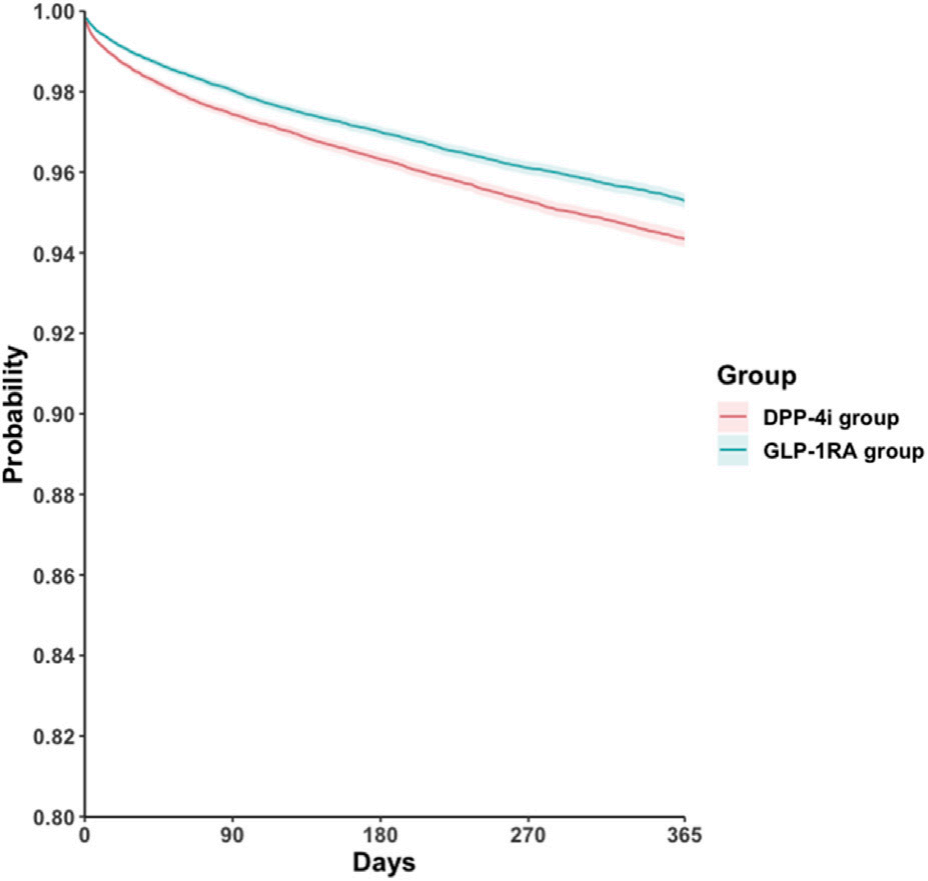
Kaplan-Meier time-to-event free curves of primary outcome comparison to GLP-1RA and DPP-4i groups. DPP-4i, dipeptidyl peptidase-4 inhibitor; GLP-1 RA, glucagon-like peptide-1 receptor agonist.

In the matched cohort of 62,267 patients in each group, EHR-ascertained SICM or SICM-related cardiac dysfunction occurred in 2,503 patients (4.0%) in the GLP-1RA group and 3,059 patients (4.9%) in the DPP-4i group, corresponding to a HR of 0.82 (95% CI 0.78–0.87; P < 0.001; E-value 1.7; Table 2). Kaplan-Meier curve showed a consistently higher event-free probability in the GLP-1RA group than in the DPP-4i group (log-rank P < 0.001; Figure 2). For secondary outcomes, GLP-1RA use was associated with a significantly lower risk of the surrogate outcome (1.5% vs. 2.1%; HR 0.70, 95% CI 0.64–0.76; P < 0.001; E-value 2.2). Reduced risks were also observed for systolic type SIC (HR 0.85, 95% CI 0.77–0.93; P < 0.001), diastolic type SIC (HR 0.88, 95% CI 0.81–0.95; P = 0.002), and hyperdynamic type SIC (HR 0.82, 95% CI 0.71–0.95; P = 0.009). No significant difference was identified for RVD type SIC (HR 0.92, 95% CI 0.71–1.20; P = 0.531). Given the relatively small number of RVD-type events and the wide confidence interval, this subgroup estimate was considered exploratory.

**TABLE 2:** Hazard ratio of outcomes between GLP-1RA and DPP-4i groups.

Outcome	GLP-1RA group (n = 62,267)	DPP-4i group (n = 62,267)	HR (95% CI)	*P* Value	E-value (95% LCL)
	Events (%)	Events (%)			
Primary outcome	​	​	​	​	​
SIC	2,503 (4.0)	3,059 (4.9)	0.82 (0.78, 0.87)	<0.001	1.7 (1.6)
Secondary outcomes	​	​	​	​	​
Surrogate outcome	906 (1.5)	1,305 (2.1)	0.70 (0.64, 0.76)	<0.001	2.2 (2.0)
Systolic type SIC	795 (1.3)	954 (1.5)	0.85 (0.77, 0.93)	<0.001	1.6 (1.4)
Diastolic type SIC	1,053 (1.7)	1,226 (2.0)	0.88 (0.81, 0.95)	0.002	1.5 (1.3)
RVD type SIC	105 (0.2)	117 (0.2)	0.92 (0.71, 1.20)	0.531	1.4 (1.0)
Hyperdynamic type SIC	324 (0.5)	401 (0.6)	0.82 (0.71, 0.95)	0.009	1.7 (1.3)
New onset heart failure	2,259 (3.6)	2,711 (4.4)	0.85 (0.80, 0.89)	0.001	1.6 (1.5)
All-cause mortality	1,921 (3.1)	3,060 (4.9)	0.64 (0.60, 0.68)	0.001	2.5 (2.3)

CI, confidence interval; DPP-4i, dipeptidyl peptidase-4, inhibitor; GLP-1, RA, glucagon-like peptide-1, receptor agonist; HR, hazard ratio; RVD, right ventricular dysfunction; LCL, lower confidence limit; SIC, sepsis-induced cardiomyopathy.

In addition, GLP-1RA use was associated with a lower risk of new-onset heart failure (3.6% vs. 4.4%; HR 0.85, 95% CI 0.80–0.89; P = 0.001; E-value 1.6) and all-cause mortality (3.1% vs. 4.9%; HR 0.64, 95% CI 0.60–0.68; P = 0.001; E-value 2.5) (Supplementary Figure S1).

#### Subgroup analysis

**FIGURE 3:**
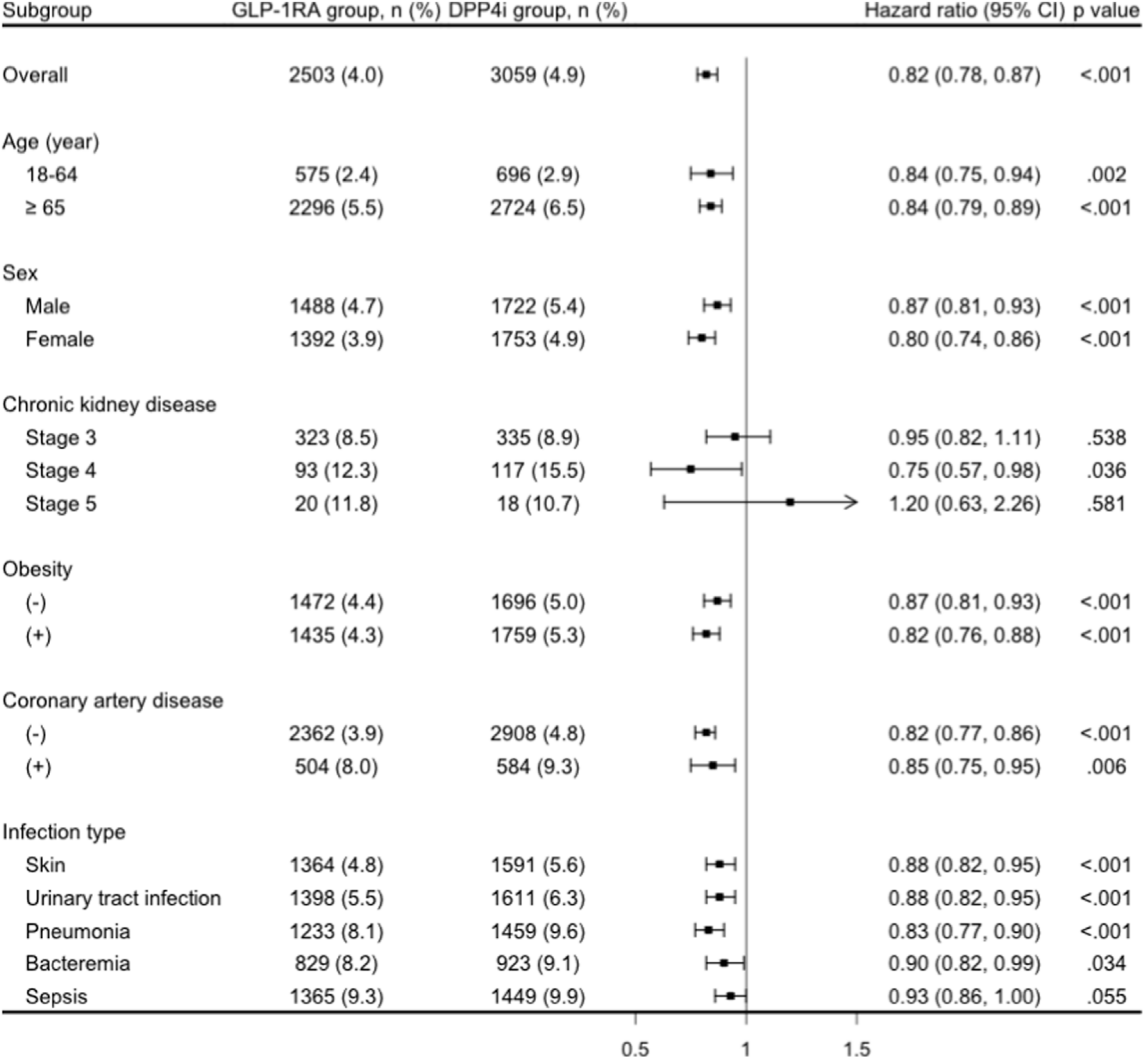
Subgroup analysis for the risk of primary outcome comparison to GLP-1RA and DPP-4i groups. CI, confidence interval; DPP-4i, dipeptidyl peptidase-4 inhibitor; GLP-1 RA, glucagon-like peptide-1 receptor agonist.

Figure 3 presents the subgroup analyses for the primary outcome. The association remained consistent across age groups. The HR was 0.84 (95% CI 0.75–0.94; P = 0.002) among patients aged 18–64 years and 0.84 (95% CI 0.79–0.89; P < 0.001) among those aged ≥65 years. Similar findings were observed in both sexes, with HRs of 0.87 (95% CI 0.81–0.93; P < 0.001) in males and 0.80 (95% CI 0.74–0.86; P < 0.001) in females. When stratified by CKD stage, the association was statistically significant in stage 4 CKD (HR 0.75, 95% CI 0.57–0.98; P = 0.036), whereas no significant difference was observed in stage 3 (HR 0.95, 95% CI 0.82–1.11; P = 0.538) or stage 5 CKD (HR 1.20, 95% CI 0.63–2.26; P = 0.581). The CKD stage 5 estimate was imprecise, with a wide confidence interval crossing unity, and should be interpreted cautiously.

Consistent associations were also observed regardless of obesity status, with HRs of 0.87 (95% CI 0.81–0.93; P < 0.001) in non-obese patients and 0.82 (95% CI 0.76–0.88; P < 0.001) in patients with obesity. Similarly, GLP-1RA use was associated with lower risk in patients with and without CAD. Across infection subtypes, the reduced risk associated with GLP-1RA use was observed for skin infection, urinary tract infection, pneumonia, and bacteremia. The association for sepsis showed a borderline significance (HR 0.93, 95% CI 0.86–1.00; P = 0.055).

#### Sensitivity analysis

In the negative control analyses, no significant association was observed between GLP-1RA use and hearing loss (HR 1.04, 95% CI 0.96–1.12; P = 0.356) or hernia (HR 0.98, 95% CI 0.93–1.04; P = 0.504; Supplementary Table S4). Landmark analyses demonstrated consistent findings with the primary analysis. From 1 day to 3 months after the index date, the HR for the primary outcome was 0.74 (95% CI 0.69–0.81; P = 0.001). Similar risk reductions were observed for the 1-day to 6-month (HR 0.79, 95% CI 0.74–0.85; P < 0.001) and 1-day to 9-month (HR 0.83, 95% CI 0.79–0.88; P < 0.001) intervals. In analyses restricted to later follow-up periods, the association remained significant from 3 months to 1 year (HR 0.88, 95% CI 0.82–0.95; P < 0.001), 6 months to 1 year (HR 0.87, 95% CI 0.79–0.95; P = 0.002), and 9 months to 1 year (HR 0.80, 95% CI 0.68–0.93; P = 0.005; Supplementary Table S5).

## Discussion

In this large United States real-world cohort study of 124,534 patients with T2DM experiencing infection, we found that pre-infection use of GLP-1 RAs, compared with DPP-4is, was associated with a lower risk of SICM over 1 year. Beyond the primary outcome, GLP-1 RA use was also associated with favorable trends in clinically relevant secondary outcomes, including surrogate outcomes, new-onset heart failure and all-cause mortality. Importantly, our study further extended existing evidence by characterizing distinct phenotypes of SICM. Notably, the observed associations were primarily driven by significant risk reductions in LVSD, LVDD, and LVHF phenotypes. The robustness of these associations was further supported by negative control outcome analyses and landmark analyses. Additionally, subgroup analyses across clinically relevant strata revealed generally consistent directions of effect, suggesting that the findings were not driven by a specific patient subgroup. However, the CKD stage 5 subgroup warrants cautious interpretation. Although the overall association favored antecedent GLP-1 RA exposure, the point estimate in patients with CKD stage 5 was in the opposite direction and was not statistically significant, with a wide confidence interval. This finding should not be interpreted as evidence of harm, but it highlights the uncertainty of the association in advanced CKD. Patients with CKD stage 5 represent a clinically heterogeneous population, with important differences in dialysis status, uremic burden, volume status, frailty, infection severity, competing mortality risk, and medication tolerability. In addition, GLP-1 RA prescribing in advanced CKD may be influenced by clinician selection, residual kidney function, gastrointestinal tolerability, nutritional status, and perceived safety concerns, all of which may contribute to residual confounding. Therefore, the subgroup result in CKD stage 5 should be considered exploratory and hypothesis-generating, and the potential cardiovascular implications of antecedent GLP-1 RA exposure in advanced CKD require dedicated investigation.

Taken together, these findings provide a coherent real-world framework to contextualize the potential role of antecedent glucose-lowering therapy in modulating cardiovascular vulnerability following infection.

Our findings build upon a growing body of literature linking infection and sepsis to subsequent myocardial injury and transient cardiac dysfunction (Frencken et al., 2018). Prior studies have consistently shown that acute infection, particularly when complicated by sepsis, is frequently accompanied by myocardial injury and reversible cardiomyopathy, which are associated with adverse short- and long-term cardiovascular outcomes (Beesley et al., 2018; Vasile et al., 2013). However, most previous investigations have focused on critically ill or sepsis-only populations, with limited attention to infection-indexed cohorts, antecedent cardiometabolic risk profiles, or heterogeneity in cardiac phenotypes. By anchoring our analysis at the time of infection and systematically characterizing SICM subtypes, our study extends these observations to a broader and clinically relevant population of patients with T2DM.

Notably, accumulating evidence from randomized trials and real-world studies has demonstrated that GLP-1 RAs confer cardiovascular benefits in patients with T2DM beyond glucose lowering (Kallash and Frishman, 2025). Emerging data further suggest that GLP-1 RA use may be associated with lower risks of systemic inflammation, and certain infection-related complications. For example, in a large real-world study including more than 330,000 patients with T2DM, GLP-1 RA use, compared with DPP-4is, was associated with substantially lower risks of incident pneumonia and severe sepsis (Henney et al., 2024). In parallel, evidence from randomized controlled trials supports a broader infection-related benefit of GLP-1 RAs, with meta-analyses demonstrating modest but significant reductions in respiratory infections and serious infections overall (Han et al., 2025; Yu et al., 2023). Collectively, these findings provide converging clinical evidence that GLP-1 RAs may favorably modulate host inflammatory and immune responses during acute illness, thereby offering biological plausibility for the observed association with reduced SICM in our study (Fan et al., 2025).

Several biological mechanisms may plausibly underlie the observed association between antecedent GLP-1 RA use and a lower risk of SICM. Acute infection and sepsis are characterized by systemic inflammation, endothelial dysfunction, microcirculatory impairment, and profound metabolic stress, all of which can precipitate transient myocardial dysfunction (Aissaoui et al., 2025; Hollenberg and Singer, 2021). Preclinical studies using endotoxemia and polymicrobial sepsis models have demonstrated that activation of GLP-1 receptor signaling can directly preserve cardiac performance under septic stress (Helmstädter et al., 2020; Ku et al., 2010). In experimental models of lipopolysaccharide-induced sepsis, treatment with GLP-1 RAs has been shown to increase myocardial cyclic adenosine monophosphate (cAMP) levels, improve myocardial contractility and blood pressure, and enhance survival, supporting a direct cardioprotective effect during systemic inflammation (Drucker, 2018). Beyond hemodynamic effects, GLP-1 receptor activation has been shown to exert anti-inflammatory and cytoprotective actions at the myocardial and vascular levels. Experimental data suggest that GLP-1 RAs attenuate proinflammatory signaling pathways, including suppression of nuclear factor-κB–mediated cytokine responses, and reduce downstream release of inflammatory mediators such as tumor necrosis factor-α and interleukins (Wong et al., 2024; Li et al., 2020). In parallel, GLP-1 receptor signaling has been linked to activation of prosurvival pathways, including cAMP/protein kinase A and phosphoinositide 3-kinase/Akt signaling, leading to reduced cardiomyocyte apoptosis, attenuation of oxidative stress, and preservation of mitochondrial function, mechanisms highly relevant to the metabolic and energetic failure observed in SICM (Zhang et al., 2016; Zhang et al., 2020). Furthermore, endothelial dysfunction and microvascular dysregulation also play a central role in the pathogenesis of SICM, and GLP-1 RAs have been shown to improve endothelial bioavailability and stabilize microcirculatory flow in experimental sepsis models (Helmstädter et al., 2020; Liu et al., 2017; Helmstädter et al., 2021). Collectively, these mechanistic data provide biological plausibility that antecedent GLP-1 RA therapy may preferentially modulate left ventricular vulnerability during acute infection, consistent with the predominant attenuation of LVSD, LVDD, and LVHF phenotypes observed in our study.

The absence of a significant association for RVD-type SICM also warrants consideration. In contrast to the LVSD, LVDD, and LVHF phenotypes, antecedent GLP-1 RA exposure was not significantly associated with RVD-type SICM. This finding suggests that the observed association may not extend uniformly across all SICM phenotypes. One possible explanation is that right ventricular dysfunction during infection or sepsis may be driven more strongly by pulmonary vascular and respiratory factors, including hypoxemia, acute respiratory distress, mechanical ventilation, fluid shifts, and increased right ventricular afterload (Vallabhajosyula et al., 2021), whereas left ventricular dysfunction may be more closely related to systemic inflammation, endothelial dysfunction, metabolic stress, and direct myocardial injury. However, these respiratory and hemodynamic variables were not consistently available in the TriNetX dataset, and the number of RVD-type events was relatively small, resulting in limited precision. Therefore, the null RVD finding should be interpreted cautiously and should not be viewed as definitive evidence of a phenotype-specific absence of association.

From a clinical perspective, our findings highlight the potential relevance of antecedent glucose-lowering therapy in shaping cardiovascular vulnerability during acute infection among patients with T2DM. Infections and sepsis are common precipitants of cardiac dysfunction and subsequent heart failure in this population, yet preventive strategies targeting SICM remain limited. Within this context, our results suggest that pre-infection use of GLP-1 RAs may be associated with a more favorable cardiovascular risk profile following infection, particularly with respect to left ventricular–predominant dysfunction phenotypes. These findings underscore the potential importance of long-term cardiometabolic optimization in patients at high risk for infection. For clinicians managing patients with T2DM who have substantial cardiometabolic burden or recurrent infectious risk, consideration of glucose-lowering therapies with established cardiovascular and anti-inflammatory properties may have implications beyond glycemic control. Our findings further support the need for heightened cardiovascular surveillance following infection in high-risk diabetic patients and suggest that SICM may represent an important intermediate phenotype linking infection to downstream heart failure and mortality.

### Limitations

This study has several limitations that merit consideration. First, the TriNetX platform is a registry-based electronic health record database, and the potential for patient misclassification or underrepresentation, particularly among individuals with milder disease severity or limited healthcare utilization, may affect the generalizability of our findings. In addition, the use of diagnostic and procedural codes to ascertain exposures, covariates, and outcomes introduces the possibility of misclassification bias. To mitigate this concern, we conducted negative control outcome analyses, which yielded null associations and thereby reduced the likelihood of systematic coding-related bias. Additionally, because this analysis was restricted to the United States TriNetX network, the generalizability of our findings to non-US healthcare systems and populations may be limited. Furthermore, although the SICM outcome definition was based on a prespecified EHR-based operational algorithm, it has not been independently validated against adjudicated critical care diagnoses. Therefore, reference to this algorithm should be interpreted as an operational ascertainment strategy rather than external validation, and residual outcome misclassification remains possible.

Second, detailed information on medication dose, treatment duration, and adherence was not available, precluding assessment of dose–response relationships and limiting evaluation of longer-term treatment effects. Third, although extensive propensity score matching was employed to balance a broad range of measured baseline characteristics, residual confounding due to unmeasured or incompletely captured variables cannot be fully excluded. To further evaluate the potential influence of unmeasured confounding, we calculated E-values. For the primary outcome, the E-value was 1.7, indicating that an unmeasured confounder associated with both antecedent GLP-1 RA use and SICM by a risk ratio of at least 1.7 could potentially explain the observed association. Fourth, SICM ascertainment in EHR data remains challenging and has not been independently validated using a standardized critical care adjudication framework. Although we excluded patients with pre-index records of heart failure, cardiomyopathy, cardiogenic shock, acute pulmonary edema, or objective cardiac dysfunction to reduce the likelihood of capturing prevalent cardiac disease, residual misclassification remains possible. In particular, ICD-10-CM codes for heart failure and cardiomyopathy are broad and may not reliably distinguish acute sepsis-related myocardial dysfunction from chronic structural heart disease. Similarly, elevated NT-proBNP and cardiac troponin I levels are not specific diagnostic criteria for SICM and may reflect myocardial stress, renal dysfunction, demand ischemia, or other acute illness-related mechanisms. Therefore, the primary outcome should be interpreted as EHR-ascertained SICM or SICM-related cardiac dysfunction rather than adjudicated SICM. Fifth, a major limitation of this study is the inability to fully account for infection severity at presentation. Severity of infection or sepsis is a dominant determinant of SICM risk, and patients with antecedent GLP-1 RA exposure may have differed from those with antecedent DPP-4i exposure in ways not fully captured by structured EHR data. Although lactate level, lactate ≥2 mmol/L, infection subtype, comorbidities, medication use, and available laboratory variables were included in the propensity-score model, lactate represents only an imperfect proxy for acute illness severity. Granular critical care variables, including SOFA score, vasopressor requirement, ICU admission, mechanical ventilation, fluid balance, source control, and antimicrobial timing, were not consistently available in the TriNetX platform. Therefore, residual confounding by infection severity cannot be excluded and may have contributed to the observed association between antecedent GLP-1 RA exposure and lower SICM risk. The findings should therefore be interpreted as hypothesis-generating associations rather than evidence of a causal protective effect.

Finally, cause-specific mortality data were not accessible within the database, preventing differentiation between cardiovascular and non-cardiovascular deaths and potentially leading to outcome misclassification.

## Conclusion

In this large United States real-world cohort of patients with T2DM experiencing infection, antecedent use of GLP-1 RAs was associated with a lower risk of SICM and favorable cardiovascular outcomes compared with an active comparator. These associations were consistent across clinically relevant subgroups and supported by multiple sensitivity analyses, including negative control and landmark analyses. While causality cannot be inferred and residual confounding by infection severity remains possible, these findings support further prospective investigation of the relationship between antecedent GLP-1 RA exposure and cardiovascular outcomes after infection.

## Data Availability

The data analyzed in this study is subject to the following licenses/restrictions: The datasets analyzed in this study were obtained from the TriNetX research network. Access to the data is restricted to participating institutions with a licensed subscription to the TriNetX platform. Only de-identified, aggregated data were available to the authors, and the datasets cannot be publicly shared because of data-use agreements and privacy restrictions. Requests to access these datasets should be directed to TriNetX Research Network Email: support@trinetx.com.
