# Supplementary material for: Antecedent glucagon-like peptide-1 receptor agonist use and risk of sepsis-induced cardiomyopathy in type 2 diabetes: a United States real-world active-comparator cohort study
Source: Front Pharmacol. 2026 Jul 31;17:1877392. doi: 10.3389/fphar.2026.1877392 (PMC13472920; doi:10.3389/fphar.2026.1877392)
Supplement: Supplementary file 1 [file Table1.docx]

**Table S1.** Demographic, diagnostic, procedural, medication, visit, and laboratory codes utilized in the definition of the cohorts.

| **Category** | **Code** | **Description** |
| --- | --- | --- |
| **Index drug** | | |
| medication | NLM:ATC:A10BH | Dipeptidyl peptidase 4 (DPP-4) inhibitors |
| medication | NLM:ATC:A10BJ | Glucagon-like peptide-1 (GLP-1) analogues |
| **Infection** | | |
| diagnosis | UMLS:ICD10CM:A00-B99 | Certain infectious and parasitic diseases |
| **T2D** | | |
| diagnosis | UMLS:ICD10CM:E11 | Type 2 diabetes mellitus |

**Table S2.** Definitions of covariates coding used in this study.

| **Code** | **Description** |
| --- | --- |
| AI | Age at Index |
| 2106-3 | White |
| F | Female |
| 2054-5 | Black or African American |
| M | Male |
| 2028-9 | Asian |
| E11.2 | Type 2 diabetes mellitus with kidney complications |
| E11.3 | Type 2 diabetes mellitus with ophthalmic complications |
| E11.4 | Type 2 diabetes mellitus with neurological complications |
| E11.5 | Type 2 diabetes mellitus with circulatory complications |
| E78 | Disorders of lipoprotein metabolism and other lipidemias |
| I48 | Atrial fibrillation and flutter |
| F17 | Nicotine dependence |
| E66 | Overweight and obesity |
| N18 | Chronic kidney disease (CKD) |
| CV100 | BETA BLOCKERS/RELATED |
| A10BA | Biguanides |
| A10BK | Sodium-glucose co-transporter 2 (SGLT2) inhibitors |
| CV800 | ACE INHIBITORS |
| CV805 | ANGIOTENSIN II INHIBITOR |
| A10A | INSULINS AND ANALOGUES |
| A10BG | Thiazolidinediones |
| 9068 | Lactate [Moles/volume] in Serum, Plasma or Blood |
| 9002 | Cholesterol in LDL [Mass/volume] in Serum or Plasma |
| 9037 | Hemoglobin A1c/Hemoglobin.total in Blood |
| 8001 | Glomerular filtration rate/1.73 sq M.predicted [Volume Rate/Area] in Serum, Plasma or Blood by Creatinine-based formula (MDRD) |
| 9083 | BMI |

**Table S3.** Definitions of outcomes coding used in this study.

| **Code** | **Description** |
| --- | --- |
| **SIC** | |
| UMLS:ICD10CM:J81.0 | Acute pulmonary edema |
| UMLS:ICD10CM:I50 | Heart failure |
| UMLS:ICD10CM:R57.0 | Cardiogenic shock |
| TNX:FINDING:2003 | Left Ventricular Ejection Fraction (LVEF) (%) (at most 50.00 % (most recent occurrence)) |
| UMLS:LNC:33762-6 | Natriuretic peptide.B prohormone N-Terminal [Mass/volume] in Serum or Plasma (at least 1800.00 pg/mL (most recent occurrence)) |
| TNX:9005 | Troponin I.cardiac [Mass/volume] in Serum, Plasma or Blood (at least 0.50 ng/mL (most recent occurrence)) |
| UMLS:ICD10CM:I42 | Cardiomyopathy |
| **New onset HF** | |
| UMLS:ICD10CM:J81.0 | Acute pulmonary edema |
| UMLS:ICD10CM:I50 | Heart failure |
| UMLS:ICD10CM:R57.0 | Cardiogenic shock |
| UMLS:ICD10CM:I42 | Cardiomyopathy |
| **All-cause mortality** | |
| Deceased | Deceased |
| UMLS:ICD10CM:R99 | Ill-defined and unknown cause of mortality |
| **Surrogate** | |
| TNX:FINDING:2003 | Left Ventricular Ejection Fraction (LVEF) (%) (at most 50.00 % (most recent occurrence)) |
| UMLS:LNC:33762-6 | Natriuretic peptide.B prohormone N-Terminal [Mass/volume] in Serum or Plasma (at least 1800.00 pg/mL (most recent occurrence)) |
| TNX:9005 | Troponin I.cardiac [Mass/volume] in Serum, Plasma or Blood (at least 0.50 ng/mL (most recent occurrence)) |
| **Systolic SIC** | |
| UMLS:ICD10CM:I50.2 | Systolic (congestive) heart failure |
| TNX:FINDING:2003 | Left Ventricular Ejection Fraction (LVEF) (%) (at most 50.00 % (most recent occurrence)) |
| **Diastolic SIC** | |
| UMLS:ICD10CM:I50.3 | Diastolic (congestive) heart failure |
| **RVD SIC** | |
| UMLS:ICD10CM:I50.81 | Right heart failure |
| UMLS:LNC:77903-3 | Tricuspid valve annulus Excursion distance during systole by US.M-mode (at most 1.70 cm (most recent occurrence)) |
| **Hyperdynamic SIC** | |
| UMLS:ICD10CM:I50.83 | High output heart failure |
| TNX:FINDING:2003 | Left Ventricular Ejection Fraction (LVEF) (%) (at least 70.00 % (most recent occurrence)) |

**Table S4**. Negative outcome control.

| Outcome | HR (95% CI) | *P* value |
| --- | --- | --- |
|  |  |  |
| Hearing loss | 1.04 (0.96,1.12) | 0.356 |
| Hernia | 0.98 (0.93,1.04) | 0.504 |

**Table S5**. Sensitivity analysis with landmark analysis of the primary outcome.

| Outcome | HR (95% CI) | P value |
| --- | --- | --- |
| 1-day to 3-months | 0.74 (0.69,0.81) | 0.001 |
| 1-day to 6-months | 0.79 (0.74,0.85) | <.001 |
| 1-day to 9-months | 0.83 (0.79,0.88) | <.001 |
| 3-months to 1-year | 0.88 (0.82,0.95) | <.001 |
| 6-months to 1-year | 0.87 (0.79,0.95) | 0.002 |
| 9-months to 1-year | 0.80 (0.68,0.93) | 0.005 |

Figure S1: Central illustration of the active-comparator exposure cohort design and main findings.
